# WITHDRAWN: Broadcasters, receivers, functional groups of metabolites and the link to heart failure using polygenic factors 

**DOI:** 10.21203/rs.3.rs-3272974/v2

**Published:** 2024-04-01

**Authors:** Azam Yazdani

## Abstract

The full text of this preprint has been withdrawn, as it was submitted in error. Therefore, the authors do not wish this work to be cited as a reference. Questions should be directed to the corresponding author.

